# Acrylamide Induces Antiapoptotic Autophagy and Apoptosis by Activating PERK Pathway in SH-SY5Y Cells

**DOI:** 10.3390/toxics13010041

**Published:** 2025-01-07

**Authors:** Yiqi Wang, Ying Liu, Xing Zhang, Yang Jiao, Lian Duan, Ruijie Cheng, Ning Yang, Hong Yan

**Affiliations:** 1 MOE Key Lab of Environment and Health, Department of Health Toxicology, School of Public Health, Tongji Medical College, Huazhong University of Science and Technology, 13 Hangkong-Road, Wuhan 430030, China; 2 Department of Environmental Health, College of Preventive Medicine, Army Medical University (Third Military Medical University), Chongqing 400038, China; 3 Department of Laboratory Medicine, Children’s Hospital, Zhejiang University School of Medicine, National Clinical Research Center for Child Health, National Children’s Regional Medical Center, Hangzhou 310000, China

**Keywords:** acrylamide, PERK pathway, apoptosis, autophagy

## Abstract

Acrylamide (ACR) is a commonly used organic compound that exhibits evident neurotoxicity in humans. Our previous studies showed that the mechanisms of ACR-caused neurotoxicity included apoptosis, PERK-mediated endoplasmic reticulum stress, and autophagy, but the relationships among them were still unclear. This paper investigated the relationships among apoptosis, autophagy, and the PERK pathway to demonstrate the mechanism of ACR neurotoxicity further. Different doses of ACR were set to value ACR toxicity. Then, a PERK inhibitor and autophagy inhibitor, GSK2606414 and 3-methyladenine (3-MA), were used separately to inhibit the PERK pathway and autophagy activation in SH-SY5Y cells under ACR treatment. With the increase of ACR dose, the apoptotic rate increased in a dose-dependent manner. After the inhibition of the PERK pathway, the activated apoptosis and autophagosome accumulation caused by ACR were alleviated. Under 3-MA and ACR treatment, the autophagy inhibition deteriorated apoptosis in SH-SY5Y cells but had no significant effect on ACR-induced PERK pathway activation; thus, PERK pathway-induced autophagy had an antiapoptotic role in this condition. This paper provides an experimental basis for exploring potential molecular targets to prevent and control ACR toxicity.

## 1. Introduction

Acrylamide (ACR) is an extensively used organic compound in many fields, such as mining, sewage treatment, and Western blot experiments. China, a rapidly developing country, is one of the largest producers and consumers of ACR; thus, humans may be exposed to ACR in the environment in several ways. In 1912, Louis Camille Maillard found that carbohydrate-rich food becomes brown under high temperatures, which was named the Maillard reaction. With the development of research on the Maillard reaction, many researchers discovered that amino acids (mainly asparagine) and reducing sugars in carbohydrate-rich food could produce ACR at high temperatures of over 120 °C [1]; thus, human ACR exposure is lifelong, which has gradually attracted many researchers’ attention. ACR causes neurotoxicity, carcinogenicity, genetic toxicity, and reproductive toxicity; these toxicities have already been proven in laboratory animals [2], but neurotoxicity is the only one confirmed in humans [3], and the specific mechanisms are still obscure. Therefore, this paper investigates the detailed mechanism of ACR neurotoxicity further.

Apoptosis is a programmed cell death mechanism controlled by genes [4]. Apoptosis is an important physiologic process that maintains cell populations and controls cell quality, and it could sacrifice specific cells to obtain better benefits to the organism during development and aging. Apoptosis is also induced as a defensive process when cells are impaired under diseases or extracellular stress [5]. Under specific pathological conditions, dysfunctional apoptosis could induce various diseases. Insufficient apoptosis causes uncontrolled cell proliferation and contributes to cancer development [6]. Many studies demonstrated that excessive apoptosis may lead to neurodegenerative diseases, such as Alzheimer’s disease and Parkinson’s disease [7]. Apoptosis has three pathways: the mitochondrial pathway (endogenous pathways), death receptor pathway (exogenous pathways), and endoplasmic reticulum (ER) pathway [8]. Our previous study revealed that ACR causes apoptosis in SH-SY5Y cells by activating the mitochondrial pathway at 2.5 mmol/L for 24 h [9], but whether ACR can also activate other pathways and its specific mechanism are still unknown.

The ER is a crucial organelle in the cell that participates in protein synthesis, and its homeostasis is important in maintaining cellular normal functions [10]. However, physiological and pathological stresses could disturb the homeostasis of ER, induce the accumulation of unfolded proteins, and lead to ERS [11]. In this process, unfolded protein response (UPR), a conserved intracellular mechanism, is initiated to maintain ER function [12]. UPR has three pathways: PERK-ATF4, ATF6, and IRE1-XBP1 [13]. However, when cells are exposed to severe and/or prolonged stress, excessive UPR activation could not restore ER homeostasis but increased the level of C/EBP homologous protein (CHOP, known as GADD153) by activating the PERK-ATF4 pathway and activated apoptosis [14,15]. In our previous study, the PERK-ATF4 pathway was activated in rat hippocampi exposed to 10 mg/kg of ACR for seven weeks [16], but its role is still unclear.

Macroautophagy, herein called autophagy, is an evolutionarily conserved intracellular degradation pathway. Autophagy is a dynamic process, including induction of autophagosomes, formation of autophagosomes, and fusion of autophagosomes and lysosomes and their degradation. Complete autophagy is also called autophagy flux. Autophagosomes could engulf specific cellular substrates that need to be recycled and delivered to lysosomes for subsequent degradation to maintain cellular homeostasis [17]. The association between autophagy and apoptosis is complicated and has been studied widely. Under certain conditions, autophagy could be activated to protect cells from death, but its activation could also be proapoptotic via damaging the mitochondria when the autophagy is excessively activated under specific circumstances [18]. Our previous studies showed that many autophagosomes were accumulated in rat cerebellum treated with 5 mg/kg of ACR for 12 months by drinking water, which indicated that autophagy was activated, while the fusion of autophagosome and lysosome was blocked [19], but the reason is still unclear.

ERS and autophagy are two mechanisms essential to maintaining cellular homeostasis and could lead to apoptosis, but the relationship between them is complex. ERS not only induces apoptosis but also activates autophagy [20], and the inhibition of the PERK pathway can also inhibit autophagy activation [21]. Autophagy activation helps to eliminate abnormal proteins and subsequently alleviates ERS activation [22]. Therefore, autophagy activation might also regulate PERK pathway-mediated ERS. However, other than ER, dysfunction of other organelles could cause autophagy, such as mitochondria and ribosomes [23,24], and whether ACR-induced autophagy is regulated by the PERK pathway is still unclear.

Our previous research revealed that ACR could cause autophagosome accumulation, activate the PERK pathway, and elevate apoptosis level in rats’ cerebella and hippocampi [16,19,25], but the relationships among them are still unknown. Therefore, this paper aims (a) to clarify the roles of the PERK pathway and autophagy in ACR-induced apoptosis in SH-SY5Y cells and (b) to demonstrate the relationships between ACR-caused PERK pathway activation and autophagy in SH-SY5Y cells. This paper investigates the neurotoxic mechanisms of ACR further and provides a potential target to prevent its toxicity.

## 2. Materials and Methods

### 2.1. Cell Culture

Human neuroblastoma SH-SY5Y cells were obtained from the Cell Bank/Stem Cell Bank of the Chinese Academy of Sciences and cultured at 37 °C with 5% CO_2_ in DMEM f12 medium with 1% penicillin/streptomycin and 10% fetal bovine serum. The medium was changed every two days, and the cells were separated into new culture bottles when they grew up to 80% of the bottle area. The SH-SY5Y cells were seeded in 96-well plates and cell culture dishes for treatment with different reagents, and the protein expressions, apoptotic rates, and cell viability were detected. The general toxicity test included five groups: 0, 0.63, 1.25, 2.5, and 5 mmol/L ACR. The PERK inhibition test included four groups: control group, GSK2606414 group (0.5 μmol/L), ACR group (2.5 mmol/L), and ACR+GSK2606414 group. The autophagy inhibition test included four groups: control group, 3-methyladenine (3-MA) group (2 mmol/L), ACR group (2.5 mmol/L), and ACR+3-MA group. All the reagents were directly dissolved in the medium to achieve the target concentrations. In the general toxicity test, the cells were incubated with an ACR-dissolved medium for 24 h. In the PERK inhibition test, the cells in the GSK2606414 group was exposed to a PERK inhibitor first for 2 h, washed gently with PBS twice, then the reagent-free medium was added. The cells in the GSK2606414+ACR group were exposed to a medium with 2.5 mmol/L of ACR for 24 h after washing. In the autophagy inhibition test, the cells were exposed to a medium with different reagents for 24 h. Then, the cell viability, protein expression, and apoptotic rate were assessed. Cell viability was evaluated via CCK8.

### 2.2. Western Blot Analysis

The SH-SY5Y cells were homogenized in RIPA buffer (Beyotime, Shanghai, China) containing protein phosphatase inhibitor and PMSF. Next, the extracts were centrifuged at the speed of 14,000× *g* for 15 min at 4 °C, and the supernatants of each sample were collected. The protein concentrations of each supernatant were evaluated using a BCA protein assay kit (Beyotime, Shanghai, China) and blended with the sample buffer subsequently at a proportion of 4:1. Proteins were separated via SDS-PAGE. Different-concentration gels were made to detect the expressions of diverse proteins. After transferring the proteins to a PVDF membrane from the gels, 5% nonfat milk was used to treat the PVDF membrane for 2 h, then different antibodies were utilized to incubate the PVDF membrane overnight at 4 °C: p-PERK (1:1000, 3179, CST, Danvers, MA, USA), t-PERK (1:1000, 3192, CST), microtubule-associated protein 1 light chain 3 (LC3) (1:1000, 12741, CST), GAPDH (1:1000, 5174, CST), Bax (1:1000, ab32503, Abcam, Cambridge, UK), ATF4 (1:1000, ab184909, Abcam), CHOP (1:1000, ab179823, Abcam), p62 (1:1000, A11250, ABclonal, Woburn, MA, USA), and Bcl2 (1:1000, 12789-1-AP, Proteintech, San Diego, CA, USA). Finally, the membrane was incubated with horseradish peroxidase-conjugated antirabbit IgG (1:20,000, E030120, EarthOx, Burlingame, CA, USA) for 2 h at room temperature and visualized with ECL liquid in a gel imaging system (GeneSnap version 7.05.02; SynGene, Cambridge, UK). The protein expression levels were all standardized against GAPDH intensity for data analysis.

### 2.3. Flow Cytometry

The apoptotic rate was detected using a AnnexinV–FITC/PI double-staining assay kit (KeyGEN BioTECH, Nanjing, China). The SH-SY5Y cells were harvested using trypsin to derive the cell suspension and evaluate the apoptotic rate. The cell suspension of each sample was washed twice with PBS, and the cells were resuspended with 500 μL of binding buffer. After adding 5 μL of Annexin V–FITC and 5 μL of PI working solution to the binding buffer, the samples were assessed using a FACScan flow cytometer (BD Biosciences, Franklin Lakes, NJ, USA) for 1 h with FlowJo software (version 7.6; FlowJo LLC, Ashland, OR, USA).

### 2.4. Statistical Analysis

All the data were analyzed in SPSS software version 12.0 (SPSS Inc., Chicago, IL, USA) and displayed as means ± S.D. An ANOVA test with LSD comparison was used to examine all of the above data. The differences were considered statistically significant when *p* < 0.05.

## 3. Results

### 3.1. ACR Increased Apoptosis Levels in SH-SY5Y Cells

**Figure 1:**
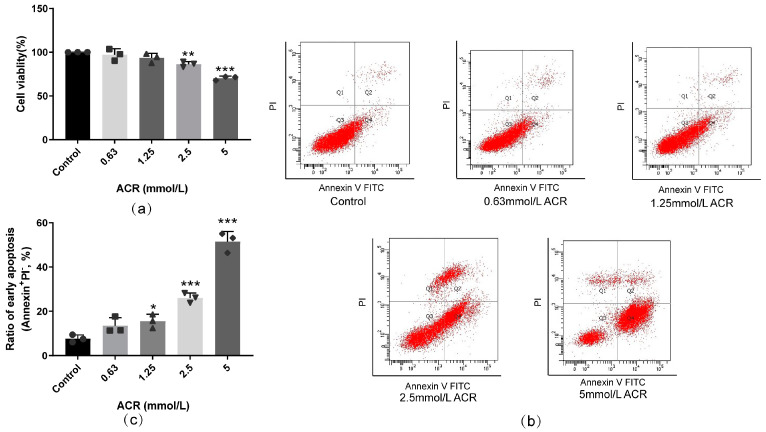
ACR treatment for 24 h caused cell death in SH-SY5Y cells. The SH-SY5Y cells were exposed to different doses of ACR for 24 h, and the cell viabilities and apoptotic rates of the SH-SY5Y cells were assessed via CCK8 (**a**) and flow cytometry subsequently (**b**,**c**), n = 3. Values are means ± S.D. * *p* < 0.05, ** *p* < 0.01, and *** *p* < 0.001 versus the control group. In this figure, circles, squares, triangles, and inverted triangles represent the data (scatter plots) of the Control group, GSK2606414 group, ACR group, and ACR+GSK2606414 group, respectively.

To assess ACR toxicity under different doses, the viabilities and apoptotic rates of the SH-SY5Y cells were measured via CCK8 and flow cytometry. The results of CCK8-manifested cell viabilities decreased with the rise in ACR doses. Compared with the control group, the cell viabilities of the SH-SY5Y cells were evidently reduced when the dose was in the range of 2.5–5 mmol/L (*p* < 0.01, Figure 1a). The apoptotic rates consistently rose with the elevation of the ACR doses. Compared with the control group, when the ACR dose was in the range of 1.25–5 mmol/L, the apoptosis level was elevated significantly (*p* < 0.05, Figure 1b,c). Thus, ACR caused cell death via apoptosis in the SH-SY5Y cells.

### 3.2. ACR-Induced Apoptosis Was Mediated by the PERK Pathway

**Figure 2:**
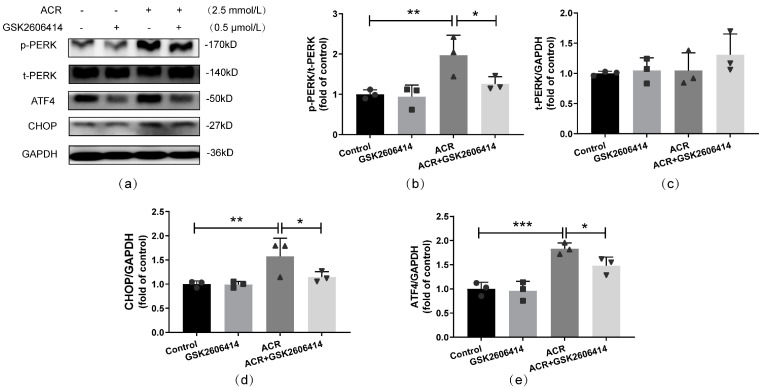
PERK-specific inhibitor, GSK2606414, inhibited ACR−induced PERK pathway activation in SH-SY5Y cells. The efficiency of GSK2606414 was evaluated via Western blot (**a**), and the corresponding quantitative analysis (**b**–**e**) is shown above. Values are means ± S.D., n = 3. * *p* < 0.05, ** *p* < 0.01, and *** *p* < 0.001. In this figure, circles, squares, triangles, and inverted triangles represent the data (scatter plots) of the Control group, GSK2606414 group, ACR group, and ACR+GSK2606414 group, respectively.

To determine the role of the PERK pathway in ACR-triggered apoptosis, the specific inhibitor of PERK, namely GSK2606414, was used to impede the activation of the PERK pathway in the SH-SY5Y cells. In this experiment, four groups were set: control group, GSK2606414 group, ACR group, and GSK2606414+ACR group. First, the efficiency of the PERK inhibitor was assessed. The results in Figure 2a–e display that the ratio of p-PERK/t-PERK and the expressions of ATF4 and CHOP in the GSK2606414+ACR group were evidently decreased compared with those of the ACR group (*p* < 0.05). Thus, the inhibitor could significantly inhibit the PERK pathway.

**Figure 3:**
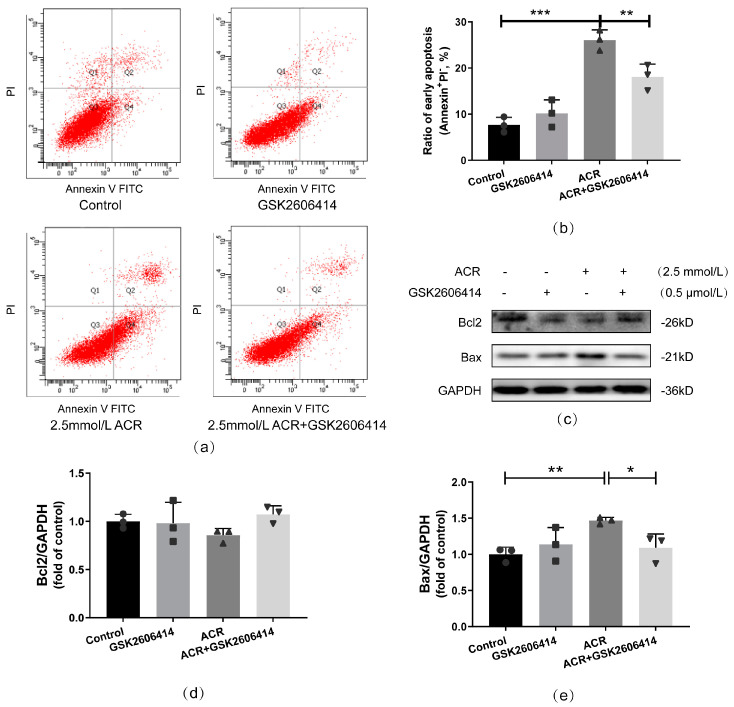
Inhibition of the PERK pathway alleviated apoptosis caused by ACR treatment in SH-SY5Y cells. The apoptotic rate and the related protein expressions were measured using flow cytometry (**a**) and Western blot (**c**), and the quantitative analyses are shown above (**b**,**d**,**e**). Values are means ± S.D., n = 3. * *p* < 0.05, ** *p* < 0.01, and *** *p* < 0.001. In this figure, circles, squares, triangles, and inverted triangles represent the data (scatter plots) of the Control group, GSK2606414 group, ACR group, and ACR+GSK2606414 group, respectively.

Then, flow cytometry and Western blot were used to measure the effects of PERK inhibition on apoptosis induced by ACR. The results of the flow cytometry indicated that increased apoptotic rates caused by the ACR treatment were reduced significantly by the PERK inhibitor in the GSK2606414+ACR group (*p* < 0.01, Figure 3a,b). The results of the Western blot consistently manifested that ACR-induced apoptosis was inhibited by GSK2606414, and the elevation of Bax was constrained by the PERK inhibitor (*p* < 0.05) but had no significant effect on Bcl2 (*p* > 0.05, Figure 3c–e).

### 3.3. Autophagy Was Prosurvival in SH-SY5Y Cells Treated with ACR

**Figure 4:**
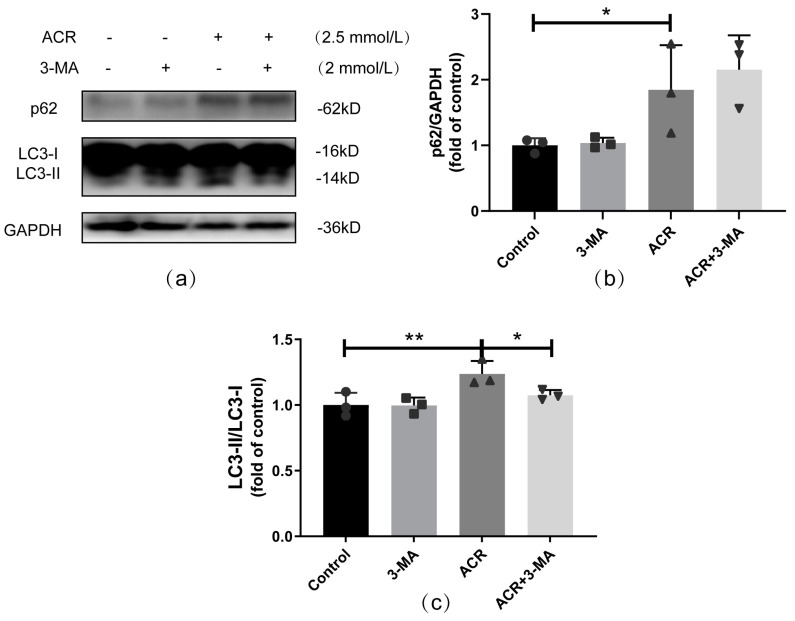
3-MA, an autophagy inhibitor, effectively inhibited ACR—caused autophagy activation in SH−SY5Y cells. The efficiency of 3-MA was assessed via Western blot (**a**), and corresponding quantitative analyses were performed subsequently (**b**,**c**). Values are means ± S.D., n = 3. * *p* < 0.05, and ** *p* < 0.01. In this figure, circles, squares, triangles, and inverted triangles represent the data (scatter plots) of the Control group, GSK2606414 group, ACR group, and ACR+GSK2606414 group, respectively.

Autophagy is an important cellular mechanism that contributes to many intracellular processes. Autophagy plays different roles under various conditions. Therefore, to determine the role of autophagy under ACR treatment, 3-MA was used to inhibit the induction of autophagosome. First, the efficiency of 3-MA was assessed by measuring the expressions of autophagy-related proteins, including LC3-II and p62, which could reflect the number of autophagosomes and the degradation level of autophagy, respectively. Compared with the ACR group, the ratio of LC3-II/LC3-I was significantly decreased in the ACR+3-MA group, but no evident difference in the p62 expression level between them was observed; thus, 3-MA could clearly inhibit autophagosome accumulation and autophagy activation under ACR treatment (*p* < 0.05, Figure 4).

**Figure 5:**
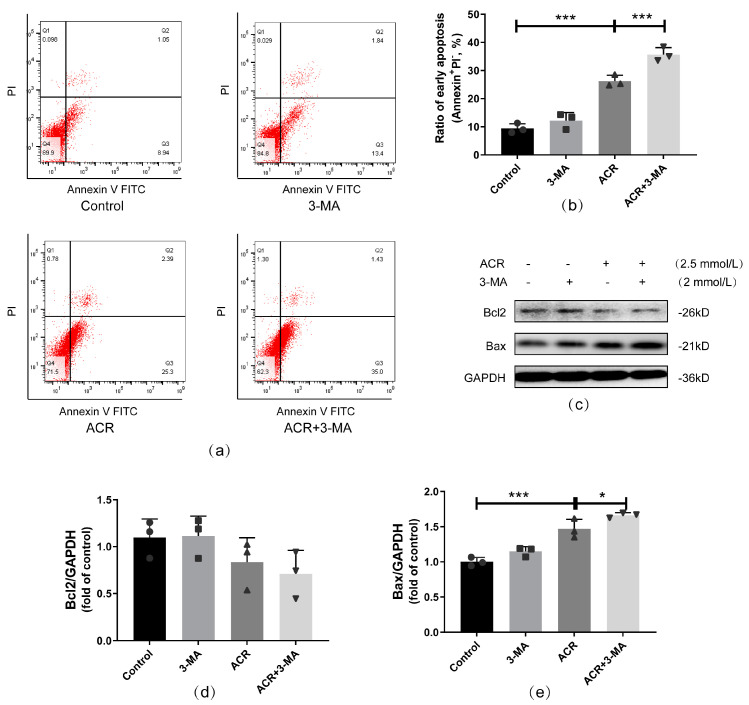
Autophagy inhibition aggravated apoptosis caused by ACR treatment in SH−SY5Y cells. Apoptotic rate and the related protein expressions were measured using flow cytometry (**a**) and Western blot (**c**), and the quantitative analyses are shown above (**b**,**d**,**e**). Values are means ± S.D., n = 3. * *p* < 0.05 and *** *p* < 0.001. In this figure, circles, squares, triangles, and inverted triangles represent the data (scatter plots) of the Control group, GSK2606414 group, ACR group, and ACR+GSK2606414 group, respectively.

Apoptosis levels were also evaluated by subsequently using flow cytometry and Western blotting. The apoptotic rate of the ACR+3-MA group was higher than that of the ACR group (*p* < 0.001, Figure 5a,b); that is, the inhibition of autophagy aggravated ACR-induced apoptosis. The results of the Western blot also indicated that the expression of the proapoptotic protein, namely Bax, increased in the ACR+3-MA group compared with the ACR group (*p* < 0.05), but no evident change was noted in Bcl2 expression (*p* > 0.05, Figure 5c–e). Thus, under ACR treatment, autophagy plays an antiapoptotic role.

### 3.4. ACR-Induced Autophagy Activation Was Controlled by PERK Pathway in SH-SY5Y Cells

**Figure 6:**
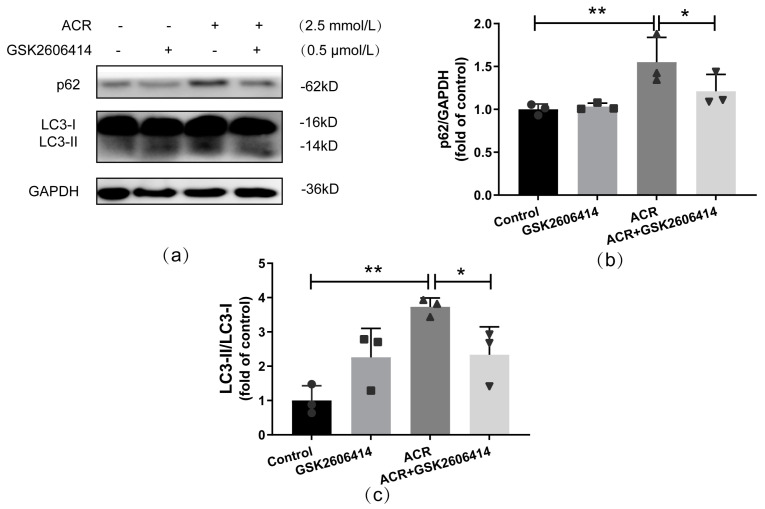
PERK pathway inhibition alleviated autophagy activation caused by ACR treatment in SH−SY5Y cells. The effects of PERK pathway inhibition on autophagy activation caused by ACR were assessed via Western blot (**a**), and the quantitative analyses are shown above (**b**,**c**). Values are means ± S.D., n = 3. * *p* < 0.05 and ** *p* < 0.01. In this figure, circles, squares, triangles, and inverted triangles represent the data (scatter plots) of the Control group, GSK2606414 group, ACR group, and ACR+GSK2606414 group, respectively.

The relationship between autophagy and PERK-mediated ERS is complex; thus, to determine the relationship between them, the autophagy condition was checked under PERK pathway inhibition. The Western blot results showed that the expressions of autophagy-related protein were clearly reduced in the GSK2606414+ACR group, which indicated that the activation of autophagy and accumulation of autophagosomes are inhibited under PERK inhibitor treatment (*p* < 0.05, Figure 6).

### 3.5. Autophagy Inhibition Had No Significant Effects on the ACR-Induced PERK Pathway Activation in SH-SY5Y Cells

**Figure 7:**
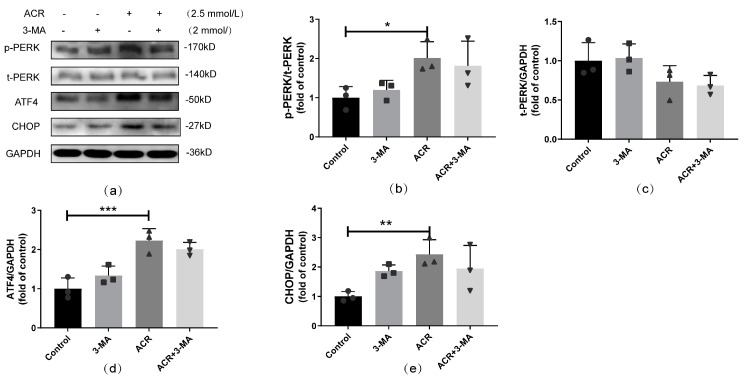
Autophagy inhibition had no significant effect on ACR−induced PERK pathway activation. The effects of autophagy inhibition on PERK pathway activation caused by ACR were assessed via Western blot (**a**), and the quantitative analyses are shown above (**b**–**e**). Values are means ± S.D., n = 3. * *p* < 0.05, ** *p* < 0.01, and *** *p* < 0.001. In this figure, circles, squares, triangles, and inverted triangles represent the data (scatter plots) of the Control group, GSK2606414 group, ACR group, and ACR+GSK2606414 group, respectively.

To determine the effects of autophagy activation on the PERK pathway under ACR treatment, autophagy was inhibited with 3-MA, then the expression level of PERK pathway-related proteins was detected via Western blot (Figure 7). Compared with the ACR group, the levels of p-PERK/t-PERK, ATF4, and CHOP in the ACR+3-MA group were not significantly changed, which further suggests that autophagy induced by ACR is mainly regulated by the PERK pathway, whereas the inhibition of autophagy has no significant effect on the ACR-activated PERK pathway.

## 4. Discussion

ACR is an extensively used chemical compound in many fields, such as paper making, mining, and Western blot experiments in laboratories. ACR causes neurotoxicity, carcinogenicity, and reproductive toxicity as revealed in animal experiments, while neurotoxicity, the only identified toxicity in humans, has been eagerly studied in populations and laboratories. According to the epidemiologic study by Liu et al., four-year ACR exposure is associated with slightly diminished cognitive ability and elevated risk of poor cognition in nonsmoking Chinese male elders [26]. Research by Guo et al. showed noticeable body weight loss and progressive impairment of motor function in rats exposed to 40 mg/kg of ACR for four weeks, which may be induced by the significantly elevated levels of TUNEL-positive cells and IL-1β and TNF-α levels in the cortex and hippocampus [27]. In our previous experiment, 12-month ACR treatment caused abnormal gait and cognitive dysfunction in SD rats by activating neuroinflammation mediated by the NLRP3 inflammasome [28], but in vitro research is needed to explore its toxicity further. In this paper, ACR-caused elevated apoptosis levels were found in SH-SY5Y cells. In this process, the PERK pathway was proapoptotic, whereas autophagy was antiapoptotic, and autophagy activation was regulated by the PERK pathway.

The neuroblastoma SH-SY5Y cell line is a subline of the SK-N-SH cell line, which has been widely used in the study of Parkinson’s disease, Alzheimer’s disease, neurotoxicity, and ischemia [29]. Although the treatment of SH-SY5Y cells with retinoic acid can enhance their differentiation, compared with undifferentiated SH-SY5Y cells, differentiated SH-SY5Y cells are less susceptible to exogenous compounds [30,31]. Therefore, in this paper, undifferentiated SH-SY5Y cells were selected to investigate the neurotoxicity of ACR. However, due to the large difference between these cells and neurons in the brain, the authors will use ACR to treat primary neurons in future work to explore the neurotoxic mechanisms of ACR further.

Apoptosis is a gene-controlled process, which could help to maintain cellular homoeostasis by removing impaired and/or unrequired cells in multicellular organisms. Apoptosis could be activated in physiological and pathological conditions. In organisms, cell division and apoptosis are balanced; once apoptosis becomes abnormal, the balance is disrupted, and diseases occur [32]. A study showed that overexpression of lncRNA PLP0P2 leads to colorectal cancer by suppressing apoptosis and promoting invasion and migration [33]. Wu et al. found that manganese exposure causes Alzheimer-like cognitive impairment, including tau hyperphosphorylation and memory deficits in male SD rats by elevating the levels of oxidative stress and apoptosis [34]. Moreover, apoptosis contributes to ACR neurotoxicity in vivo and in vitro. Research performed by our group showed that ACR treatment causes apoptosis in PC12 cells and rat cerebella [19,35], but the detailed mechanisms still need further investigation. In this paper, ACR-caused apoptosis was mediated by the PERK pathway and autophagy. ACR activates apoptosis mainly by upregulating the expression levels of Bax protein, but it has no significant effect on the levels of Bcl2. Bax and Bcl-2 are important proteins in cell apoptosis. Bax is an effector protein that can induce apoptosis by increasing the mitochondrial outer membrane’s permeability and releasing cytochrome c, whereas Bcl2 could bind to Bax and inhibit the effects of Bax. ACR could increase the expression levels of Bax but had no evident effect on bcl-2, which suggests that the change in Bax expression levels induced by ACR is not mediated by bcl-2. Studies found that p53 can directly induce the transcription of Bax [36], and the translation of p53 protein is regulated by the PERK pathway [37]. Okuno et al. found that ACR can also induce p53 protein expression in SH-SY5Y cells [38]. Therefore, the authors of this paper hypothesized that ACR might mediate the translation of p53 protein via the PERK pathway, lead to increased levels of Bax protein, and finally activate apoptosis. To verify this hypothesis, the authors constructed p53-knockout SH-SY5Y cells to explore the relationships among ACR-induced PERK pathway activation, cell apoptosis, and p53 protein.

The ER, a primary intracellular organelle in cells, is a closed 3D intertwined tubular system, and its homeostatic maintenance is important to cell survival [39]. When intracellular and/or extracellular conditions change, ER homeostasis is interrupted, causing the accumulation of unfolded proteins in the ER lumen, which may lead to cellular dysfunction. To survive, ERS and UPR, self-protective mechanisms, are initiated [40]. Some studies have shown that ERS contributes to the occurrence and development of many clinical diseases; for example, the inhibition of the ERS-mediated apoptosis pathway could alleviate the learning and memory ability of mice with Alzheimer’s disease [41]. UPR has three branches, namely PERK, IRE1, and ATF6, that help eliminate abnormal proteins and restore ER function [42]. According to Komoike, ACR activates the PERK pathway in SH-SY5Y cells but had no evident influence on the IRE1 pathway, which indicated that the PERK pathway plays a major role in ACR-induced ERS activation [43]. The PERK pathway is one of the key pathways in the UPR, which could alleviate mRNA translation, decrease protein synthesis, and help to reduce the protein load in the ER lumen, which is already stressed [44]. Therefore, when ERS is caused by changes in the internal and external environment, the PERK pathway could be activated in the early stage of stress. However, prolonged, harsh ERS would elevate CHOP expression levels and transform ERS from prosurvival to proapoptotic [45,46]. Our previous study also showed ACR activates the PERK pathway in the rat hippocampus and cortex under 10 mg/kg of ACR treatment for seven weeks [16]. Although ACR could activate the PERK pathway, its role is still unclear. In this paper, the PERK-specific inhibitor GSK2606414 hindered the apoptosis caused by ACR treatment, which indicates that the PERK pathway plays a proapoptotic role.

Autophagy is a genetically conserved lysosome-dependent mechanism in cellular homeostatic maintenance. Autophagy is essential in discarding damaged, unnecessary cellular components, which may protect organisms against aging, infection, and neurodegenerative diseases [47]. LC3 is an autophagy-associated protein in the cytoplasm and has two forms: LC3-I and LC3-II. LC3-I would be modified to LC3-II to participate in growing autophagosomes; therefore, LC3-II levels could be used to evaluate the autophagy state and the amount of autophagosomes. However, autophagy works as a double-edged sword in cells. Under normal circumstances, autophagy works as a cellular defense mechanism at low basal level in all cells to update and remove abnormal proteins and organelles, and proper intracellular and extracellular stimuli would elevate the autophagy level to protect cells from death [48]. Liu et al. found that ROS could activate autophagy through the ATM-CHK2-TRIM32-ATG7 axis to maintain cell homeostasis under metabolic stress [49]. However, excessive activation of autophagy causes cell damage; specifically, intensified, chronic autophagy leads to programmed cell death, which is also known as autophagy death or type 2 cell death [50]. Wang et al. found that autophagy is induced in PC12 cells under acute ACR [51], but the role played by autophagy under this condition is still unknown. In this paper, 3-MA was used to inhibit autophagy to moderate ACR-induced autophagy flux blockage in SH-SY5Y cells. Autophagy inhibition aggravated ACR-induced apoptosis, which suggests that autophagy protected the cells from apoptosis under ACR treatment.

The relationship between autophagy and ERS has been investigated many times, but the relationship varies under diverse conditions. Many studies have demonstrated that the PERK pathway is important in mediating the interaction between ERS and autophagy. Chen et al. found that porcine reproductive and respiratory syndrome virus caused autophagy to alleviate cell stress by activating the PERK pathway [52]. Moreover, many studies have shown that autophagy activation is not only controlled by ERS but also mediated by other factors. Wei et al. treated MIN6 cells with 4 μmol/L NaAsO_2_ for 24 h and observed increased oxidative stress level, ferroptosis, and activated MtROS-dependent autophagy [53]; this indicated that autophagy could be activated not only by ERS but also by mitochondria dysfunction. Not only does ERS regulate autophagy, but the activation of autophagy may also mediate ERS [54]. In addition, recent studies found that autophagy can be involved in regulating the activation of ERS [55]. Therefore, to determine the relationship between PERK-mediated ERS and autophagy under ACR treatment, GSK2606414 and 3-MA were used in this work to inhibit the PERK pathway and the formation of autophagosomes. After inhibiting the PERK pathway, autophagy activation was significantly inhibited in vitro, but the effects of autophagy inhibition on the PERK pathway were not significant. These results suggest that autophagy is mediated by the PERK pathway of ERS under our experimental conditions. However, whether the alleviation of autophagy flux blockage could affect PERK pathway activation under ACR treatment remains unclear. In addition, ERS and autophagy are dynamic processes and might change depending on exposure time. Therefore, in future work, ACR will be used to treat SH-SY5Y cells for different times to explore further the association among autophagy, apoptosis, and PERK pathway-mediated ERS induced by ACR under varied times of treatment.

## 5. Conclusions

The conclusions of this paper are as follows: (a) In SH-SY5Y cells, 24 h ACR treatment induces apoptosis, PERK-mediated ERS, and autophagy. (b) ACR-caused PERK pathway activation is proapoptotic and could mediate autophagy activation in SH-SY5Y cells. (c) The autophagy induced by ACR is antiapoptotic in SH-SY5Y cells. However, this paper has limitations. The ACR treatment time was short, and acute exposure can only be mimicked in vivo. In the future, a longer treatment time would be used to imitate chronic ACR exposure in vivo to investigate ACR chronic toxicity further. In addition, whether moderation autophagy flux blockage can alleviate ACR-caused PERK pathway activation still needs to be explored further.

## Data Availability

Data are available upon request. The data are not publicly available due to confidentiality and privacy considerations.
